# TRANSPORTATION BARRIERS TO ACCESS HEALTH CARE FOR SURGICAL CONDITIONS IN MALAWI a cross sectional nationwide household survey

**DOI:** 10.1186/s12889-019-6577-8

**Published:** 2019-03-05

**Authors:** Carlos Varela, Sven Young, Nyengo Mkandawire, Reinou S. Groen, Leonard Banza, Asgaut Viste

**Affiliations:** 1 Department of Surgery Kamuzu Central Hospital, Lilongwe, Malawi; 2 University of Malawi, College of Medicine, Lilongwe, Malawi; 3 Department of Clinical Medicine and Centre for International Health, University of Bergen, Bergen, Norway; 4 Department of Orthopaedic Surgery, Haukeland University Hospital, Bergen, Norway; 5 Department of Research & Development, Haukeland University Hospital, Bergen, Norway; 6 Department of Gynecology and Obstetrics, Johns Hopkins Medicine, Baltimore, USA; 7 Department of Obstetrics and Gynecology, Alaska Native Medical Centre, Anchorage, USA; 8 Department of Surgery, Queen Elizabeth Central Hospital, University of Malawi, College of Medicine, Blantyre, Malawi

**Keywords:** Transport, Barriers, Health, Access, SOSAS, Survey

## Abstract

**Background:**

It is estimated that nearly five billion people worldwide do not have access to safe surgery. This access gap disproportionately affects low-and middle-income countries (LMICs). One of the barriers to healthcare in LMICs is access to transport to a healthcare facility. Both availability and affordability of transport can be issues delaying access to health care. This study aimed to describe the main transportation factors affecting access and delay in reaching a facility for health care in Malawi.

**Methods:**

This was a multi-stage, clustered, probability sampling with systematic sampling of households for transportation access to general health and surgical care. Malawi has an estimated population of nearly 18 million people, with a total of 48,233 registered settlements spread over 28 administrative districts. 55 settlements per district were randomly selected for data collection, and 2–4 households were selected, depending on the size. Two persons per household were interviewed.

The Surgeons Overseas Assessment of Surgical need (SOSAS) tool was used by trained personnel to collect data during the months of July and August 2016.

Analysis of data from 1479 households and 2958 interviewees was by univariate and multivariate methods.

**Results:**

Analysis showed that 90.1% were rural inhabitants, and 40% were farmers. No formal employment was reported for 24.9% persons. Animal drawn carts prevailed as the most common mode of transport from home to the primary health facility - normally a health centre. Travel to secondary and tertiary level health facilities was mostly by public transport, 31.5 and 43.4% respectively. Median travel time from home to a health centre was 1 h, and 2.5 h to a central hospital. Thirty nine percent of male and 59% of female head of households reported lack financial resources to go to a hospital.

**Conclusion:**

In Malawi, lack of suitable transport, finances and prolonged travel time to a health care centre, all pose barriers to timely access of health care. Improving the availability of transport between rural health centres and district hospitals, and between the district and central hospitals, could help overcome the transportation barriers to health care.

## Background

Over the last decade the burden of disease treatable by surgery has been assumed to be arround 11%, based on an expert opinion estimate by Debas et al. in 2006 (Debas H, Gosselin R. et al. 2nd Washington World Bank; 2006 p.1245–1259) Recent estimates indicate that this figure is likely to be much higher. Estimates of the global burden of surgical disease based on a multinational survey from the provider perspective indicate that 32.9% of deaths and 28.1% of disability-adjusted life years (DALYs) could be lost due to surgically treatable conditions (Shrime et al) [1].

In rural sub-Saharan Africa there is limited information available of morbidity and mortality due to surgical diseases. In East African rural communities it has been estimated that mortality from injury related surgical conditions and cancer is 100/100,000 and 60/100,000 population per year respectively [2]. Taira et al. [3] concluded that there is very limited access and availability of surgical care across the developing world. A study in Tanzania showed that more than 90% of the population in the North of the country does not have access to orthopaedic surgical care [4].

The Lancet commission on Global Surgery estimated that 5 billion people do not have access to safe surgery [1], and the access is inequitably distributed. Lin et al. [5] showed that 96% of their study patients experienced a barrier to surgical care. 73% reported that this was due to costs, 8.2% reported that it was due to lack of a provider. They concluded that barriers to surgery were predicted by patients’ wealth and home location in The Republic of Congo [5].

Malawi, a sub-Saharan low income country (LIC), is no exception from this situation. Hospitals in rural sub-Saharan Africa, including Malawi, have not met the surgical needs of the population they serve, which has resulted in significant morbidity and mortality [6].

Though it has been shown that the health work force is severely inadequate at different levels of health provision in Malawi [7], there are multiple other barriers faced by rural communities to access health care for different medical and surgical conditions. Some of the many delayed presentations of disease seen in medical practice in low income settings have been shown to be due to the cost of transportation and time taken to reach the health facility [8], but so far there has been no study on factors posing a barrier to surgical care in Malawi.

The Malawi health care system has two main service providers: Non-governmental district facilities under Christian Health Association of Malawi (CHAM) and governmental facilities. CHAM is a network of church-owned health facilities, hospitals and training colleges run by faith based organisations with financial support from Malawi Government. CHAM has 175 health facilities and provides 37% of Malawi’s healthcare services. Unlike the free services provided by the government facilities, CHAM offers its services at a cost. The government health structure is designed in a three-tiered network of medical facilities. The primary tier is a network of rural health facilities, referred to as health centres, which are run by medical assistants and nurses, with no doctors. Further, medical supplies are often scarce. The second tier is the district hospital which caters for the critical medical case which cannot be handled in a health centre. These facilities are located centrally in each of Malawi’s 28 districts. District hospitals can handle some surgical cases if doctors or clinical officers with some surgical training are available, but they have no specialist surgeons to provide advanced surgical care. The top tier is the tertiary central hospitals which have more advanced equipment, medical supplies and medical personnel including different specialised doctors. The tertiary hospitals are only located in the four main urban areas. While CHAM provides 37% of health care delivery in Malawi, the government health facilities are responsible for 60, and 3% is served by private institutions and organisations [9].

People living in rural Malawi face major challenges due to long distances between the community and the nearest health facility, be it a health centre, district hospital or central hospital. 50% of Malawians live within 5 km from a health facility [9, 10]. Terrain and lack of road infrastructure can be a challenge in itself in some areas. Some areas are accessible by push bicycle, bicycle ambulances, and motor cycles only, others by ox-carts, lorries and motor cars. The transportation means are challenging, and in some areas the most used mode of transport is the bicycle ambulance for transferring especially maternity patients from rural health facility to district hospitals [11]. Occasionally transportation from primary health facility to secondary or tertiary health facility is provided by public hospital ambulances. Most of the roads in rural Malawi are dirt roads and many do not have bridges, making use in the rainy season even more of a challenge.

Even when the roads are passable by different means of transport, the distance from the community to the health facility has in itself been shown to influence health seeking behaviour in Malawi resulting in a gap in the accessibility of the public health service [12].

Transportation cost and cultural factors have also been shown to influence access to health care [11, 13]. Poverty and financial constraints influence decisions on where and when to seek help for health complaints [11]. Financial barriers to surgical care can either be direct or indirect. Direct costs are those directly related to care, such as surgical fees, drugs and other medical supplies, transport to health facility and hospital stay. Indirect costs are accumulated as a result of the sickness or absence from work, with loss of income and productivity [9], both for the patient, and guardians and family supplying care in the hospital and at home after discharge.

In 2016 a cluster randomised national household survey was carried out using the Surgeons Overseas Assessment of Surgical need tool (SOSAS) to estimate the burden of untreated surgical disease in Malawi [14]. We found that 35% of the population were living with a condition that was in need of a surgical consultation or intervention and that 24% of reported deaths in the preceding 12 months could have been due to a surgical condition [14]. The purpose of this study was therefore to investigate and describe factors affecting access to health facilities for general medical and surgical care in Malawi, with special focus upon transport, finances and travel time.

## Methods

### Setting

Malawi has an estimated population of 18.4 million, with a GDP per capita of USD 300 (World Bank Group data base 2016). It is estimated that only 9% of health facilities have adequate staff to implement the WHO defined Essential Health Package (EHP) [9]. Malawi has 3 regions; The Northern, Central and Southern Regions. The Central and Southern regions are the most densely populated with 6.4 and 6.8 million respectively [14]. Malawi has 28 districts, of which one, Likoma, is an island in Lake Malawi. The country has a total of 48,233 registered settlements and the vast majority of these are in the rural areas. About 90% of the population live in rural areas and are dependent mostly on subsistence farming and small scale businesses [7].

### Study design

This was a multi stage, clustered, probability sampling cross-sectional study, with systematic sampling of participants at the household level. The sample size was estimated at 1487 households based on a pilot study that was carried out in rural areas of the capital city, Lilongwe, in 2016 [14].The sample size for the individuals was estimated at 2994 individuals (95% CI) with a design effect of 1.5 at 25% prevalence of unmet surgical need in reference to the prior LMICs region reports [14–16].

The National Statistics Office provided a list of enumeration areas from the Malawi Census Board for 2008 national census records. There were 48,233 recorded settlements identified as potential enumeration areas. These settlements were randomised through computer generated random numbers, selecting 55 settlements as enumeration areas from each district in Malawi for this survey. Two or four households were systematically selected in each settlement depending on size. Two households were selected in a settlement with less than 10 households, while 4 households were selected in larger settlements. The systematic household selection was based on a floor bottle spin and selecting the third or fifth house in the direction of spin depending on the size of the settlement. Subsequently the bottle spinning was repeated after the household interview to select the next household in the new direction of the spin. The next fifth household was then picked if in a larger settlement or third household if in a smaller settlement, then repeating the process again to select the next household. Two household members were selected and interviewed per household, by first interviewing the head of the household, then selecting another member at random using random numbers based on number of members in the household. If this household member selected was a child (age below six years),mute or for some reason cannot speak, then the guardian was interviewed using the assent form on their behalf with permission granted by them. The total number of included households was 1479, with a total of 2958 people interviewed [14]. The study started out with 1486 households. Two heads of households refused to participate and for 5 households there were no data.

### Survey instrument

The Surgeons Overseas Assessment of Surgical need tool (SOSAS) was used to collect data [14–16], and. This is a questionnaire based tool with three components. The first component outlines the general house-hold information i.e. Household size, type, gender and age distribution and demographics i.e. location (urban, rural or slum). The second and third part focused on the different modes of transportation they used in the past 12 months to travel with a sick or injured household member from home to the nearest health facility, to the district hospital and to a tertiary centre, occupation of participants etc. The time taken to reach the health facility was also recorded. Inquiry on the source and availability of money by the household head to reach the health facility in the last visit was done. It also had inquiries on the assumed cost of transport (Local currency) to reach these three levels of health care, and on the different reasons that contributed to the individuals not going to the health facility in time. These were based on household level, as reported by the head of the household.

The questionnaire was installed on 17 tablet computers (iPad 2, Apple Inc.), using File Maker Pro 12.0v3 (File maker inc., USA) software for data collection in English.

### Data collection

Data collection was done by medical students trained at the end of their third year of academic training. They all underwent five days of training on how to use the questionnaire and computer tablet. A pilot study was carried out in Lilongwe prior to roll out of the main study in April 2016, to test the survey tool. Training was done prior to this pilot study and as a refresher after the pilot study in preparation for the national survey [14].

There were 32 trained data collectors, 14 female and 18 male. The period for data collection was from 1st July to 30th August 2016, during the main holiday for the medical students who did the data collection. Data collection was spilt into 2 phases. The first phase involved half of the data collectors covering all identified enumeration sites in the northern part of the Central Region and the whole of Northern Region. The second phase involved coverage of the rest of Central region and the Southern Region. In some of the enumeration areas people belonged to smaller population groups with unique languages or dialects. In these cases local translators were hired to secure good communication between the interviewer and the household member [14].

Each data collector covered approximately 4 households per day, therefore 60–64 households were interviewed every day. Interviews were done in the interviewees private homes. The collected data was merged and exported from the tablets into an Excel (Microsoft 2010) data base at the end of each day. Data was checked and exported directly into the pooled data base on a computer saver at the end of each of day, for data security and to assure the quality of the data collection [14]. Data backup was saved in cloud storage using wireless internet connection.

### Statistical analysis

Data analysis was done using SPSS version 24. Univariate and multivariate statistical analysis was done for descriptive, relative frequencies and percentages as presented in the tables.

Pearson chi square test was used to compare the availability of financial resources between males and females for travel to access surgical health care at different levels of health care provision.

## Results

Analysis performed on 1479 households revealed that 90.1% of the study population was located in rural areas (Table 1). The most common occupation among the participants was subsistence farming (40%). 24.9% of the population reported not to be formally employed by an organisation i.e. non skilled and not on a payroll by government or non-governmental organisation for monthly salary (Table 1). Home makers i.e. those who are hired on short term contracts or piece works for a few days to weeks for construction of infrastructure represented 8%, while domestic workers i.e. those who are hired to clean homes and take care of young children in the homes represented 1.9% (Table 1).

**Table 1:** Demographics and household information

	Frequency	(%)
House hold data		
Location		
Rural	1332	90.1
Urban	107	7.2
Slum	13	0.9
Not stated	27	1.8
Median household size (range)	*6 (1-47)*	
Total households	**1479**	
Individual data (Employable age group 15-66 years)	2448	
Sex distribution		
Male	977	39.9
Females	1465	59.8
Not stated	6	0.3
Median age, years (range)	34 (15-66)	
Occupation		
Farmer	979	40.0
Unemployed (non-skilled/no salary)	610	24.9
Own business	437	17.9
Home maker (house Builder)	196	8.0
Non-Government employee	95	3.9
Government employee	67	2.7
Domestic helper (House maid)	47	1.9
Not stated	17	
Total number of people surveyed	2448	0.7

It was reported by the household head that the most commonly reported mode of transport from home to the primary health facility was animal pulled carts (44.8%) while travel to secondary level and tertiary level health facility was by public transport inclusive of public hospital ambulances (31.5 and 43.4% respectively) (Table 2). About a fifth (19.1 and 19.7%) of the respondents would have to travel to their health centre or local hospital by using multiple means of transportation, with only 9.5% of using multiple modes to travel to a central hospital (Table 2). This may, for example, involve walking on foot to a certain place, hiring a push bicycle and possibly end up in a minibus or on the back of a lorry to the health facility.

**Table 2:** Transport mode to health facility for 1479 households

Mode of transport	Health centre	District hospital	Central hospital
	N (%)	N (%)	N (%)
Public transport/ambulance	50 (3.4)	466 (31.5)	642 (43.4)
Private hire	17 (1.1)	153 (10.3)	165 (11.2)
Motorcycle	26 (1.8)	34 (2.3)	2 (0.1)
Bicycle	153 (10.3)	125 (8.4)	17 (1.1)
Boat	2 (0.1)	2 (0.1)	–
Animal (Ox) cart	663 (44.8)	216 (14.6)	12 (0.8)
Walking on foot only	26 (1.8)	11 (0.7)	9 (0.6)
Multiple (Combined)	283 (19.1)	292 (19.7)	140 (9.5)
Not stated	285 (19.3)	180 (12.2)	492 (33.3)

*n* = 1479 Households

* responses from household members in reply to the question: “What is the main way for you or your household members to go to a secondary health facility?”

Regardless of mode of transportation, it took a median of 1 h to travel to the nearest health centre, 1.5 h to travel to district hospital and 2.5 h to travel to the nearest central hospital respectively.

Of the people interviewed, significantly more women than men denied having money for transport to visit health centre, district hospital and central hospital as reported by the household head (Table 3). For the household heads; 54% male and 41.6% female respondents reported to have money available to go to their local health centre, 52.8% males and 43.9% females % to district hospital and 54.5% males and 41.7% females to central hospital respectively (Table 3). At the overall household interview with family heads, less women than men stated that they had financial resources to access healthcare (*p* < 0.001).

**Table 3:** Availability of money for transportation to health care facility for 1479 households

	Local health centre			District hospital			Central hospital		
	Male Head	Female Head	Not stated	Male Head	Female Head	Not stated	Male Head	Female Head	Not stated
Yes (Y)	135(54.0%)	104 (41.6%)	11 (4.4%)	143 (52.8%)	119 (43.9%)	9 (3.3%)	85 (54.5%)	65 (41.7%)	6 (3.8%)
No (N)	185 (36.2%)	296 (57.9%)	30 (5.9%)	277 (38.8%)	394 (55.3%)	42 (5.9%)	214 (37.8%)	315 (55.7%)	37 (6.5%)
Not stated	315 (43.9%)	362 (50.4%)	41 (5.7%)	215 (43.4%)	249 (50.3%)	31 (6.3%)	336 (44.4%)	382 (50.5%)	39 (5.2%)
Chi-sq M/F:	Y/N	*p* < 0,001			*p* < 0,001			*p* < 0,001	

* as judged by the senior household member/household head interviewed in reply to the question: “…Are you always able to provide these means for transport of a sick household member……?”

Of the interviewed households heads; 56.3 and 27% would have to spend less than 1 US$ to travel to their health centre, and district hospital whereas 9.5% would spend less than 1 US$ to go to their central hospital. (Table 4). Travel to central hospital would cost up to US$ 7 in 20.6% of the respondents and only 1.1% of the same amount to travel to a health centre.

**Table 4:** Cost to reach health facility in 1479 households

	Health Centre	District hospital	Central hospital			
	N	%	N	%	N	%
US$ 0.00–0.99	832	56.3	399	27.0	141	9.5
US$ 1.00–1.50	135	9.1	292	19.7	73	4.9
US$ 1.51–3.50	63	4.3	285	19.3	161	10.9
US$ 3.51–7.00	16	1.1	159	10.8	305	20.6
US$ 7.01–13.50	5	0.3	32	2.2	153	10.3
US$ 13.51–25.00			17	1.0	38	2.6
Not stated	428	28.9	295	19.9	608	41.1

US$ 1.00 = Mk 740

Malawi GDP per capita is US$300

* as estimated by the person interviewed in reply to the question: “What does it cost you to provide transportation to a tertiary health facility for a sick household member?………”

## Discussion

This study confirmed the cost of and access to transport as significant barriers to accessing timely health care by rural communities in Malawi. Almost half of the respondents had financial constraints influencing their access to health care for surgical conditions. Inadequate road infrastructure, finances, ambulance services and expensive private transport all play a role as transportation barriers to access a health facility. This is in line with findings from another Malawian study by Abiiro et al. that people from rural areas spend more time travelling than those in urban regions, and that lack of transport, inadequate financial resources and poor road conditions limit the possibilities of people in rural communities to access health facilities [17].

Other reviews on the barriers to surgical health care encountered by patients in rural sub-Saharan Africa have identified cultural, structural, and financial constraints as barriers and concluded that patient and community education and transport needs should be made available [4, 18]. Punchak et al. reported in-adequacy in neurosurgical service in sub-Saharan Africa. The average percentage of the population with access to neuro surgery within 2 h window was shown to be 25.26% in sub-Saharan Africa, while is was 93.3% in Eastern Europe and Central Asia. This was attributed to low numbers of neurosurgery providers, equipment challenges and unreliable access to transportation to neurosurgical centres in Low- and middle income countries, including the sub-Saharan region [19].

In general, district hospitals provide less surgical care than central hospitals. Galukande et al. reported relatively low rates of major surgery at district hospitals in Eastern Africa, ranging from 50 to 450 surgical procedures per 100,000 population [18]. More than 95% of the population in South Asia and Central, Eastern and Western sub-Saharan Africa do not have access to surgical care, whereas less than 5% of the population in Australia, high-income America and Western Europe lack access [21]. This demonstrates the unmet surgical need and confirms the barriers to access to essential surgery in rural districts in sub-Saharan Africa [20].

50% of the Malawi population live within five kilometres from their health centre, a walkable distance for a healthy person, though not necessarily for someone seeking health care [9, 10]. Accordingly, it is surprising that only 1.8% of the respondents stated that travel on foot was the main way for their household members to go to a primary health facility, reflecting mostly the use of bicycles and Ox-carts for travelling short distances.Secondary level and tertiary level health facilities, however, are often far from people’s homes. District hospitals support health centres with ambulance services to ferry sick people from the health centre to the district hospital, and on to the tertiary central hospitals if needed. This hospital ambulance transport support system usually gives priority to maternity patients, especially urgent obstetric complications. Each district hospital has a minimum of 15 health centres to support within its catchment area, and may have only 2 or 3 ambulances. Unfortunately, these may also be off the road due to lack of fuel or vehicle spare parts, and they often fail to go to health centres to fetch patients.

Eventually, communities have to find alternative transportation from home to the health centre, and also from the health centre to a district hospital. Elderly people may carry children on their back over considerable distances just to seek surgical health care. Sometimes when they get to a health centre, they are informed that the facility does not have resources for surgical treatment e.g. sutures for closing wounds or plaster of Paris (POP) for treating fractures, hence they have to wait for an ambulance coming for obstetric emergency patient to come and collect them together to go to the district hospital. Otherwise they should be prepared to find and pay for alternative means of transportation to travel to the district hospital. This time consuming activity contributes to the delay in presentation to health facility as another barrier.

Central hospitals offer tertiary surgical services for emergency and elective conditions, but are located in the big cities far from most rural areas. These central hospitals serve as both secondary and tertiary level facilities due to inadequate surgical services at the secondary level. Sometimes resources are so limited that health service seekers have to bypass the district hospitals and travel directly to the central hospital in order to be assisted accordingly. When patients get to a district hospital, they get referred to a central hospital, and usually the mode of transport is by public hospital ambulance which sometimes delays time of travel because they have to wait to fill it up with more patients, hence posing as another barrier.

Government public transportation is not available in most rural areas either due to poor road infrastructure or unavailability of public transport supported by government. This has led to private owned sources of transport which are also very costly. This private mode of transportation varies from ox-cart, push bicycle, motorcycles, and motor-tricycles to big lorries, minibuses and buses. Depending on availability of funds, and unpredictability of availability of ambulance services, the only way is to hire an ox-cart, push bicycle or any other mode of transportation to ferry them to hospital. Though it costs less than USD 1 to travel to a health centre, the needed financial resources are often not readily available. About 56% of the population reported to have an equivalent of 1 USD to enable them to travel to a health centre.

White et al. [22] had similar findings of cost of travel to centres that provide surgical care and have recommended that NGOs and other stake holders should be involved in LMICs provision of surgical care by actively engaging in case finding and offer surgical services [22]. This should be done by travelling to the rural location to provide surgical care rather than expecting the patients to travel to access care [23]. This could be achieved by designing surgical camps in the district and upscaling surgical services at district hospital level.

The transport barriers described in this study contribute significantly to inadequate access to general medical and surgical services in Malawi such that the burden of disease is still unacceptalby large, with as many as 35% of the population living with a condition needing a surgical consultation or treatment (Varela et al) [14]. Though human and other resources contribute to this burden, transport availability between health centres and district hospitals and between district hospital and central hospital also needs to be emphasised in order to improve health for all Malawians, especially surgical health care as a non-communicable disease section of the essential package.

One of the limitations to this study was that the transport limitation was not assessed according to seasonal availability of funds in the communities. In Malawi, during harvest time of farm produce, the availability of money is better than during the field preparation and growing season for example. This study was done during the time farmers were busy preparing their fields for the upcoming growing season. This could be associated with low availability of funds to support transport to hospital because priority is on farm inputs like seed and fertiliser for the farms. Another limitation was that the cost of mode of transport was not estimated in reference to the type of transport used. For example the cost of ox-cart was not easily compared to cost of push bicycle or car because the cost of transportation is based on the distance travelled not the vessel used. Cars and buses are generally more expensive that the other modes of transportation though not readily available. One weakness of using the tool for data collection was that this was based on interviews and hence has a recollection and recall bias may have influenced the results. Also, the information was self-reported and lacked objective measures. Missing information in many reported variables is in general a major concern, although there is no reason to believe that this has introduced a selection bias. The strengths of this study are the size, systematic selection of households and participants, use of a standardized instrument, use of trained personnel and high response rate.

## Conclusion

In Malawi, lack of suitable transport, lack of finances and prolonged travel time to tertiary health facilities, all pose barriers to timely access of health care. Improving the availability of free transport for patients between rural health centres and district hospitals, and between the district and central hospitals, could help overcome some of the most pronounced barriers to surgical health care experienced by people in rural Malawi.

## Abbreviations

CHAM: Christian Health Association of Malawi
CI: Confidence Interval
DALYs: Disability Adjusted Life years
EPH: Essential health Package
GDP: Gross Domestic Product
LIC: Low Income countries
LMIC: Low and Middle Income countries
NGO: Non-Government Organisations
NORAD: Norwegian Agency for Development
NORHED: Norwegian Programme for Capacity Development in Higher Education and Research for Development
POP: Plaster of Paris
SOSAS: Surgeons Overseas Assessment of Surgical need
SPSS: Statistical Package for Social Scientists
USD: United States Dollar
WHO: World Health Organisation
